# Geospatial Analysis of Malaria and Typhoid Prevalence Due to Waste Dumpsite Exposure in Kinshasa Districts with and Without Waste Services: A Case Study of Bandalungwa and Bumbu, Democratic Republic of Congo

**DOI:** 10.3390/ijerph21111495

**Published:** 2024-11-11

**Authors:** Yllah Kang Okin, Helmut Yabar, Karume Lubula Kevin, Takeshi Mizunoya, Yoshiro Higano

**Affiliations:** 1 Degree Programs in Life and Earth Sciences, Graduate School of Science and Technology, Doctoral Program in Environmental Studies, University of Tsukuba, Tsukuba 305-8577, Japan; 2 Faculty of Life and Environmental Sciences, University of Tsukuba, Tsukuba 305-8577, Japan; 3 Faculté des Sciences, Département de Biologie, Université de Kinshasa, Kinshasa H8J5+6PX, Congo; 4 University of Tsukuba, Tsukuba 305-8577, Japan

**Keywords:** geospatial analysis, public health impacts, exposure to waste dumpsites, municipal solid waste, Kinshasa

## Abstract

Municipal solid waste (MSW) management poses substantial challenges in rapidly urbanizing areas, with implications for both the environment and public health. This study focuses on the city of Kinshasa in the Democratic Republic of Congo, investigating whether the presence or absence of solid waste collection services results in varying health and economic impacts, and additionally, seeking to establish a correlation between residing in proximity to dumpsites and the prevalence of diseases like malaria and typhoid, thereby providing a comprehensive understanding of the health implications tied to waste exposure. Health data were collected through survey questionnaires, and the geospatial distribution of 19 dumpsites was analyzed using Google Earth Pro 7.3.1 for satellite imagery and GIS software 10.3.1 to map dumpsites and define 1 km buffer zones around the largest dumpsites for household sampling. Statistical analyses were conducted using R Version 4.2.3, employing Chi-square tests for disease prevalence and logistic regression to assess associations between waste management practices and health outcomes. A multivariate regression was used to evaluate correlations between discomfort symptoms (e.g., nasal and eye irritation) and waste activities. The geospatial analysis revealed significant variation in dumpsite size and location, with larger dumpsites near water bodies and flood-prone areas. The study contributes valuable insights into waste-related health risks, emphasizing the need for improved waste management policies in rapidly urbanizing areas like Kinshasa. The socio-demographic analysis reveals distinct traits within the surveyed populations of two communes, Bandalungwa (Bandal) and Bumbu. Bumbu, characterized by larger open dumpsites and limited waste collection services, exhibits a higher prevalence of certain diseases, particularly typhoid fever, and malaria. This discrepancy is statistically significant (*p* < 2.2 × 10^−16^), suggesting a potential link between waste exposure and disease prevalence. In Bandal, self-waste collection is a high risk of exposure to typhoid (OR = 4.834 and *p* = 0.00001), but the implementation of a waste collection service shows protective effect (OR = 0.206 and *p* = 0.00001). The lack of waste collection services in Bumbu increases the risk of exposure, although not significantly (OR = 2.268 and *p* = 0.08). Key findings indicate that waste disposal methods significantly differ between Bandal and Bumbu. Bumbu’s reliance on burning and dumping creates environments conducive to disease vectors, contributing to elevated disease transmission risks. However, an in-depth correlation analysis reveals that specific waste management practices, such as burning, burying, and open dumping, do not exhibit statistically significant associations with disease prevalence, underlining the complexity of disease dynamics. This study contributes valuable insights into the importance for urban public health, particularly in rapidly urbanizing cities like Kinshasa, where inadequate waste management exacerbates health risks. By investigating the correlation between proximity to unregulated dumpsites and the prevalence of diseases such as malaria and typhoid fever, the research underscores the urgent need for targeted waste management policies. The stark health disparities between Bandal, with better waste services, and Bumbu, where services are lacking, highlight the protective effect of organized waste collection. These findings suggest that expanding public waste services and enforcing stricter regulations on waste disposal could reduce disease prevalence in vulnerable areas. Additionally, the study supports integrating waste management into urban planning as a critical public health measure. Its evidence-based approach offers valuable insights for policymakers in Kinshasa and other cities facing similar challenges, emphasizing the broader health implications of environmental governance in urban settings.

## 1. Introduction

Waste management stands as a critical challenge for urban areas worldwide, profoundly affecting public health and environmental well-being [1]. Globally, cities face growing challenges in handling the surge in solid waste due to increasing populations and economic activity. This problem is more severe in low- and middle-income countries, where urban waste management systems tend to be underdeveloped. In Africa, only 55% of the annually generated 125 million tons of municipal solid waste (MSW) is collected [2]. This amount reduces further to 44% in sub-Saharan Africa [3]. It is not uncommon to observe small and large dumping sites nearby habitations across the African continent [4]. Over 90% of the produced waste is disposed of in open dumpsites and uncontrolled landfills [5], and 19 of the world’s 50 biggest dumping sites are located in this region [5]. Population growth is one of the major drivers for waste generation increase [6]. Compared to other urban agglomerations, the population in African cities has been growing rapidly in recent years, with a 150% increase between 2000 and 2015 [7] and the trend is projected to nearly double by 2050 [8]. This situation calls for implementing adequate MSW management systems to tackle numerous public health concerns and the increasing negative environmental impacts [5].

The improper disposal of waste at these sites can lead to the release of hazardous substances, including potentially toxic elements (PTEs) and other pollutants, into the environment. These pollutants, once released, can contaminate soil, water, and air, leading to a range of adverse health effects. Previous studies, such as the “Feasibility of Energy Recovery Potential of Municipal Solid Waste in Northwest Iran” [9], have highlighted the environmental and health risks associated with waste dumpsites. Improper disposal of solid waste, particularly through illegal dumpsites, also poses a significant threat to public health by creating a potential link between these environmental concerns and the incidence of various infectious diseases [10,11]. Inadequate waste management practices can lead to the accumulation of stagnant water in discarded items, creating ideal breeding grounds for disease-carrying vectors such as mosquitoes. This stagnant water becomes a reservoir for the proliferation of malaria-transmitting mosquitoes, thereby increasing the risk of malaria transmission in nearby communities [12,13]. Furthermore, the decomposition of organic waste in these dumpsites can contaminate surrounding soil and water sources with pathogens, contributing to the spread of waterborne diseases such as typhoid. The unsanitary conditions associated with illegal dumpsites attract rodents and insects that serve as carriers of various infections [14,15].

Solid waste-related environmental pollution, is a well-recognized catalyst for a range of health issues, including waterborne diseases like typhoid, malaria, and cholera, alongside non-communicable diseases such as cancer and asthma [16,17,18,19]. These adverse health impacts are particularly pronounced in low-income countries, where nearly 90% of deaths are attributed to pollution, with air and water pollution being the primary culprits [1,5,7,20]. As economies rapidly develop, they grapple with new forms of pollution, predominantly stemming from chemicals and pesticides [21]. These pollutants, which may arise from various human activities, including ecosystem-altering interventions, manifest as air, water, land, energy related, and a diverse array of chemical substances [10,22]. Urbanization and industrialization introduce a spectrum of Substances of Concern (SoC) into the environment, affecting public health [23]. For instance, air pollution encompasses particulates, sulfur oxides, nitrogen oxides, hydrocarbons, and carbon monoxide, with contaminants like carbon emissions and lead being common in urban areas [10]. In parallel, water pollution gives rise to waterborne diseases, including typhoid, amoebiasis, and ascariasis [24,25]. The family of potentially toxic elements (PTEs), such as mercury, lead, and cadmium, are significant contributors to pollution, with their accumulation in ecosystems threatening both human and animal life [10]. Biological pollutants, often stemming from human activities, encompass viruses, bacteria, and various pathogens. Of particular concern among pollutants are PTEs due to their non-degradability, persistence, and toxicity [10,26,27], with substantial impacts on ecosystems and human health, as they infiltrate the human body through diverse pathways such as skin absorption, food, air, soil and water [10,28].

In the sprawling capital of the Democratic Republic of Congo, Kinshasa, the proliferation of waste dumpsites has emerged as a prominent concern [29]. Extensive research has illuminated the health and economic consequences borne by communities in proximity to these dumpsites [17,30]. Studies have elucidated the adverse health outcomes, including respiratory issues, skin ailments, and gastrointestinal infections [31,32]. The close proximity to waste sites also raises the incidence of illnesses like malaria, typhoid, cholera, dengue, yellow fever, and hepatitis [17,30,31,32,33]. This is primarily because waste sites create environmental conditions that are conducive to the breeding of disease-carrying mosquitoes, flies, and gastrointestinal pathogens [34]. Notably, in Africa, approximately 23% of vector-borne, diarrheal, and cardiovascular diseases can be attributed to environmental factors [35].

Malaria and typhoid fever are prevalent health concerns in the Democratic Republic of Congo (DR Congo) and particularly in its capital city, Kinshasa. The DR Congo carries a significant burden of both diseases [36], with malaria being a major public health challenge due to the country’s tropical climate and the presence of the Anopheles mosquito, which transmits the malaria parasite [37]. The prevalence of malaria is notably high in many regions of the country, including Kinshasa, making it one of the leading causes of morbidity and mortality, especially among children and pregnant women, accounting for 44 percent of all outpatient visits and for 22 percent of deaths in 2018 [38]. The DR Congo ranks as the second highest in the world for both malaria cases and fatalities. In 2021, 12.3% of global malaria cases and deaths, along with 12.6% of all malaria-related deaths, were attributed to the DRC. Additionally, the country represented 53% of malaria cases in Central Africa during that year [38]. During 1986–1987, the average malaria prevalence in six districts within Kinshasa stood at 50%, with a notably higher prevalence observed in the outlying districts [36]. This trend mirrored the dispersion of the primary malaria vector, Anopheles gambiae, which exhibited lower presence in the city center compared to the peripheral areas [36].

Typhoid fever is another noteworthy health issue in Kinshasa and the broader DR Congo with repeated and sometimes severe outbreaks in the country [39]. This bacterial infection is typically spread through contaminated food and water sources, and its incidence is often associated with inadequate sanitation and hygiene practices [39]. In urban areas like Kinshasa, where access to clean water and proper sanitation can be limited, typhoid outbreaks are not uncommon. From 1 October to 10 December 2004, in Kinshasa, there were 615 severe cases of peritonitis, either with or without perforation, and among these cases, there were 134 fatalities, resulting in a case fatality rate of 21.8%. Among the 32 samples tested, 5 of them were found to be positive for S. Typhi [40]. In 2009, the province of Kinshasa reported 118,727 cases of typhoid fever. With an estimated population of 10,000,000 residents in Kinshasa, the corresponding incidence of typhoid fever for 2008 was 1662 cases per 100,000 people. This incidence rate was significantly higher than what is typically expected for a country in Africa [41]. The prevalence of typhoid underscores the importance of improving water and sanitation infrastructure, as well as enhancing public health education to reduce the risk of infection. 

Illegal dumpsites exert a range of detrimental impacts that extend beyond public health concerns. One significant consequence is the heightened risk of flooding in nearby areas. When water channels become clogged with improperly discarded waste, drainage systems are compromised, leading to increased flood susceptibility during heavy rainfall [42]. Additionally, the unsightly appearance of illegal dumpsites negatively affects the properties values and the aesthetic appeal of the surrounding environment, impairing the natural beauty of landscapes and diminishing the overall quality of life for residents [43,44]. This aesthetic degradation, in turn, has adverse effects on local tourism, deterring visitors and potential economic opportunities for affected communities [45]. Furthermore, the presence of illegal dumpsites contributes to neighborhood degradation, fostering a sense of neglect and disarray that can lead to increased crime rates. The accumulation of waste provides cover for illicit activities and encourages the deterioration of social cohesion, amplifying the potential for criminality [46]. Moreover, the pervasive environmental degradation linked to illegal dumpsites has been associated with psychological distress, including increased rates of depression and anxiety among residents [47,48]. 

Addressing the negative impacts of illegal dumpsites is a multifaceted concern that underscores the importance of proper waste disposal practices in safeguarding public health and preventing the outbreak of preventable diseases. This issue requires concerted efforts in waste management, public awareness, policy enforcement and evidence-based decision making to mitigate the environmental and health risks associated with improper solid waste disposal.

This research, focusing on the spatial distribution of waste open dumpsites in Kinshasa, particularly in the communes of Bumbu and Bandalungwa (Bandal), investigates whether the presence or absence of public services results in varying health and economic impacts. 

The study tests two main hypotheses: (1) areas with public waste collection services will have lower incidences of diseases such as malaria and typhoid compared to areas without these services; (2) proximity to waste dumpsites is associated with a higher prevalence of malaria and typhoid, with residents closer to dumpsites expected to experience higher disease rates.

Additionally, the study seeks to establish a correlation between residing in proximity to dumpsites and the prevalence of diseases like malaria and typhoid, providing a comprehensive understanding of the health implications tied to waste exposure.

The aim of this research is to demonstrate the critical importance of waste management services in safeguarding public health and enhancing environmental quality. By providing evidence on the positive effects of waste management services, the study seeks to influence decision makers to prioritize and invest in waste management infrastructure. Additionally, the research aspires to support evidence-based decision making, demonstrating how proper waste management not only improves health outcomes but also benefits the economy. Ultimately, the goal is to drive informed policy changes that can lead to better waste management practices and improved quality of life in Kinshasa and similar urban areas.

### Previous Works on Public Health Impacts of Inadequate Waste Management

Inadequate waste management practices can have dire consequences for public health. Studies have documented increased disease transmission, respiratory problems, and other health risks associated with exposure to waste dumpsites. Epidemiological investigations and health surveys are vital tools for identifying these health impacts, linking waste exposure to health outcomes, and informing policy decisions [17,30,31,32,33,49,50,51].

The following Table 1 offers insights into the public health implications stemming from inadequate waste management practices, particularly from exposure to waste dumpsites. The studies discussed here employ epidemiological analyses and health surveys to quantify and qualify health risks and establish links between waste exposure and adverse health outcomes, emphasizing the critical importance of effective waste management for public well-being.

- Main Agreements and Disagreements in the Literature

Within the domain of public health impacts stemming from inadequate waste management, researchers concur on the existence of health risks associated with waste exposure. Numerous studies [17,30,32,33,34,49,50,51] demonstrate that exposure to waste dumpsites can lead to increased health risks, including higher disease transmission rates, higher multiplication of vectors and an elevated incidence of respiratory problems. 

However, disagreements in the literature are prominent, largely centered around the complexities of assessing health impacts. The primary point of contention relates to the non-standardized methodologies used across studies. Different researchers employ varying methodologies and data collection techniques, making it challenging to draw direct comparisons and generalize findings. The result is a diverse body of research that often reaches inconsistent conclusions, thus impeding the development of universally applicable guidelines [52].

Another significant source of disagreement lies in the challenge of linking health outcomes solely to the presence of waste dumpsites. Factors such as the duration and timing of exposure to waste, as well as the determination of the exact zones or populations exposed, are critical but complicated to ascertain accurately [53]. These complexities contribute to variations in reported health impacts and hinder the establishment of causal relationships between waste exposure and health outcomes.

b. Overall Perspective

The overall perspective drawn from the literature is that the public health impacts of inadequate waste management are a crucial concern, particularly in densely populated urban areas. While there is a consensus on the existence of health risks associated with waste exposure, the non-standardized methods and difficulties in drawing direct correlations pose substantial challenges. 

This study attempts to reduce the insufficiency in the methodology by ensuring a statistically representative sample in the survey and uses spatial analysis to geolocalize potential impacted zones by waste dumpsites more accurately, in hope that this further contributes to the academia in the field of public health and waste management.

c. Gaps and Areas for Further Research

Addressing the disagreements and complexities in assessing public health impacts, further research should focus on the standardization of methodologies and data collection techniques. Establishing common guidelines for research on waste exposure and health outcomes would facilitate more robust comparisons across studies. Additionally, there is a need for comprehensive investigations into the duration and timing of exposure to waste and the determination of exposed zones or populations. The development of standardized protocols for assessing health impacts related to waste exposure in specific contexts, such as Kinshasa, D.R. Congo, is essential for accurate risk assessments and the development of effective public health interventions.

## 2. Materials and Methods

### 2.1. Study Area 

The geospatial analysis and evaluation of health impacts due to exposure to waste dumpsites were conducted in the city of Kinshasa, the capital of the Democratic Republic of Congo (DR Congo) with the following characteristics:

- Geographical Setting: Kinshasa is located in the southwestern part of DR Congo, along the Congo River. The city’s topography is relatively flat, with some gentle undulations. This geographical setting influences the dispersion of pollutants and waste.
- Climate: Kinshasa experiences a tropical rainforest climate, characterized by high temperatures and humidity throughout the year. Average temperatures range from 22 °C to 31 °C (72 °F to 88 °F). The city has a pronounced wet season from October to May, with frequent and heavy rainfall, which can impact the management and dispersion of waste.
- Wind Direction: The prevailing winds in Kinshasa are generally from the west, influenced by the city’s proximity to the Congo River and the surrounding topography. This wind pattern can affect how pollutants from waste dumpsites are dispersed across different areas of the city.
- Distance from Residential Areas: In Kinshasa, the proximity of waste dumpsites to residential areas varies significantly. Some dumpsites are located relatively close to densely populated neighborhoods, which can exacerbate health risks. The spatial distribution of these sites is influenced by urban planning.
- Urban Infrastructure: Kinshasa’s urban infrastructure is a mix of planned and informal development. While some areas benefit from organized waste collection and management services, many others rely on informal or insufficient waste disposal methods.
- Population Density and Distribution: Kinshasa is one of Africa’s most populous cities, with a rapidly growing population. The population density varies across the city, with some communes experiencing high density and others more sparsely populated.
- Environmental Concerns: The combination of high temperatures, humidity, and heavy rainfall in Kinshasa creates an environment where waste degrades rapidly, potentially leading to increased exposure to harmful pollutants. The interaction between climatic factors and waste management practices is crucial for understanding health impacts.

**Figure 1:**
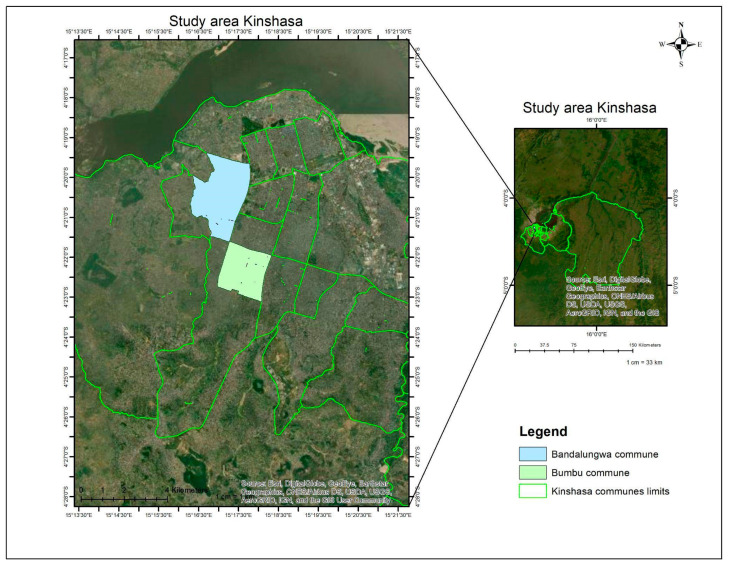
Map of study areas in Kinshasa.

Kinshasa is administratively divided into 24 communes, of which 9 benefit from formally established waste collection services (Table 2). The study focused on two specific communes within Kinshasa: Bumbu and Bandal [Figure 1].

Bandalungwa, home to 362,766 people in an area of 6.8 km^2^, is a well-established and planned commune in Kinshasa, benefiting from formal waste collection services. The area consists of older houses made from durable materials and is divided into seven neighborhoods. In terms of education, Bandalungwa has 42 nursery schools, 63 elementary schools, and 35 secondary schools, which indicates a relatively well-developed educational infrastructure. The commune is also better served in terms of water supply services: 65% of the population has direct access to house connections, 35% can access a neighboring water supply tap, and 0% of the population lacks access to any water supply service. Bandalungwa is located within the Lukunga district, one of the six districts of Kinshasa, and represents approximately 3.02% of Kinshasa’s total population of 12 million (as per 2017 estimates) [54,55]. Bumbu, with a population of 536,018 and an area of 5.3 km^2^, is one of the southern suburbs of Kinshasa that does not have formal waste collection services, resulting in significant environmental and health challenges. The commune features newer houses built from durable materials and is divided into thirteen neighborhoods. Educational facilities in Bumbu include 41 nursery schools, 77 elementary schools, and 52 secondary schools, reflecting a growing but unevenly distributed educational infrastructure. Water supply services in Bumbu are more limited compared to Bandalungwa: 39% of the population has access to house connections, 23% can access a neighboring water tap, and 38% have no access to any water supply service. These conditions, coupled with a lack of formal waste collection services, could contribute to the commune’s lower living standards and heightened health risks. Bumbu is located in the Funa district, and it accounts for approximately 4.47% of Kinshasa’s total population [54,55].

The rationale for selecting Bandal is that it represents the most densely populated commune that benefits from an established formal waste collection service. The choice of Bumbu is based on its status as a planned urban center with a substantial population and is known to contain multiple dumpsites as identified in previous research field surveys.

**Table 2:** Demographic and socio-economic characteristics of communes in Kinshasa [55].

Area type	Commune	Population (2017)	Area (km^2^)	Collection Services
Residential Area	Gombe	80,696	29.3	Yes
	Limete	466,113	67.6	No
	Ngaliema	1,147,924	224.3	No
Old City	Kintambo	179,581	2.7	Yes
	Barumbu	172,449	4.7	Yes
	Kinshasa	152,778	2.9	Yes
	Lingwala	148,534	2.9	Yes
New City	Ngiri-ngiri	167,019	3.4	Yes
	Kasa-vubu	114,152	5	Yes
Planned City	Kalamu	287,045	6.6	Yes
	Lemba	505,836	23.7	No
	Matete	343,584	4.9	No
	Bandalungwa	362,766	6.8	Yes
	N’djili	651,007	11.4	No
Southern Suburbs	Ngaba	279,329	4	No
	Selembao	471,504	23.2	No
	Bumbu	536,018	5.3	No
	Makala	329,725	5.6	No
Urban Periphery	Kisenso	579,147	16.6	No
	Masina	1,070,858	69.7	No
	Kimbanseke	1,678,395	237.8	No
	Mont-ngafula	714,074	358.9	No
	N’sele	772,027	898.8	No
	Maluku	1,294,439	7948	No

### 2.2. Dumpsites Data Collection

In January 2023, a comprehensive process was followed to collect data on waste dumpsite locations and volumes. The first step involved obtaining research authorization from the University of Kinshasa. This authorization, which included details on the research timeline and locations, was addressed to the authorities of Bandalungwa and Bumbu communes and stamped by the Faculty of Sciences at the university. Each authorization document was then submitted to the city hall of the respective commune, where it required the signature and agreement of the mayor regarding the research period and locations. With these signed documents in hand, the research team proceeded to the field, starting in Bandalungwa and then moving to Bumbu.

During fieldwork, the team encountered challenges from individuals claiming to be controlling certain areas. Despite showing the official documents, some of these individuals demanded money to allow the research to continue. To mitigate these issues, the team opted to either change their location or return on different days, ensuring that the research could proceed without compromising integrity.

Data collection involved using GPS devices and measuring tapes to accurately record the locations and volumes of dumpsites. These data points were cross verified with Google Earth satellite imagery to ensure accuracy. Geographic Information Systems (GISs) were employed to map the distribution of the dumpsites, and for each commune, the largest dumpsite was selected. A 1 km buffer zone around each large dumpsite was established based on a thorough review of similar studies [17,30,32,33,34,49,50,51]. Remote sensing techniques were used to monitor the expansion and changes in waste accumulation over time, providing insights into shifts in waste management practices within the city. This validation process ensured the accuracy and reliability of the collected dumpsite data.

### 2.3. Survey Sample Sizing 

The Yamane formula, named after the Japanese statistician Satoru Yamane, is often used to determine the appropriate sample size for a research study. It helps researchers determine the number of subjects or data points they need to collect from a larger population to ensure that their findings are statistically valid and reliable.

The Yamane formula [56] for sample sizing is as follows:

(1) $$n=\frac{N}{1+Ne^{2}}$$

where

“*n*” represents the required sample size.
“*N*” represents the total population size.
“*e*” represents the margin of error.

Based on the data presented in Table 1, population figures for both communes are available. Given that this study considers households rather than individuals, statistically representative sample sizes for each commune was calculated, considering an average household size of 6 people in Kinshasa city [57].

### 2.4. Health Impact Assessment

Health data were collected through survey questionnaires, and the geospatial distribution of 19 dumpsites was analyzed using Google Earth Pro for satellite imagery and GIS software 10.3.1 to map dumpsites and define 1 km buffer zones around the largest dumpsites for household sampling. 

Self-reported recurrent diagnosed illnesses and symptoms were determined through the survey questionnaire. The questionnaire’s content underwent a thorough review by public health experts from the University of Kinshasa. The same questionnaire was separately implemented in both communes from March 2023 to August 2023. A letter of authorization to survey was obtained from the university of Kinshasa and validated by the mayor of each commune.

**Figure 2:**
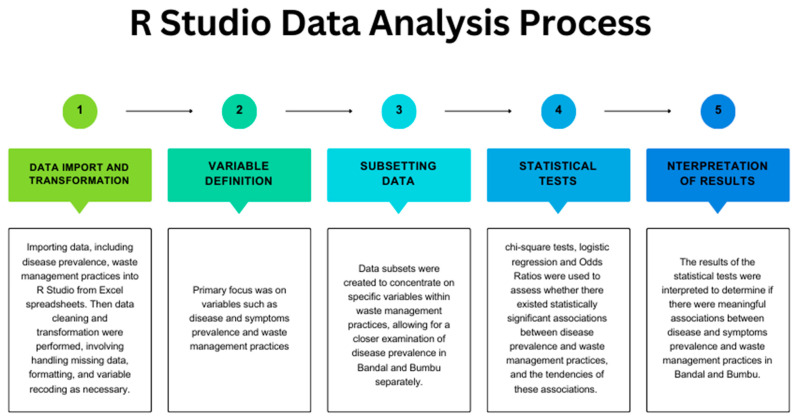
R studio data analysis process.

Statistical analyses were carried out using R Version 4.2.3. The Chi-square test was used to calculate the statistical prevalence difference between Bandal and Bumbu for malaria and typhoid fever. Logistic regression was used to test potential association between disease prevalence and waste service, and self-collection separately. Additionally, multi-regression analysis tests the correlation of activities involving trash with discomfort (nasal irritation, eye irritation) felt by the surrounding population. All the statistical tests performed were conducted in order to compare Bandal and Bumbu’s waste management strategies [Figure 2]. 

## 3. Results and Discussions

### 3.1. Dumpsites Mapping 

**Figure 3:**
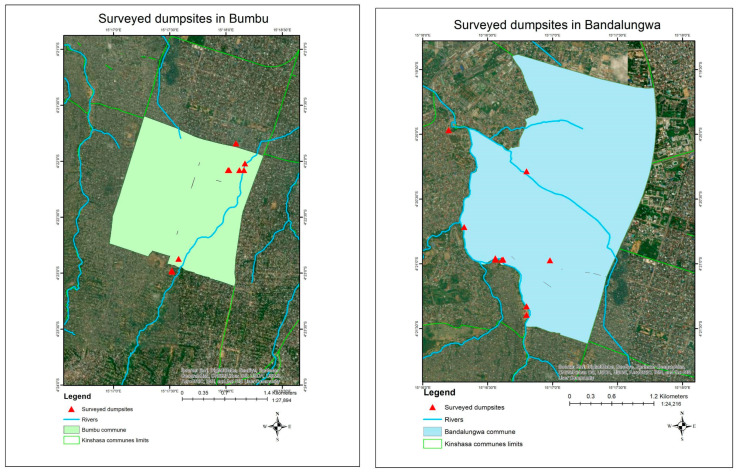
Surveyed dumpsites in Bumbu and Bandal.

A total of 19 dumpsites were identified in both communes, with 9 dumpsites in Bumbu, and 10 in Bandal [Table 3] [Figure 3]. 

The dumpsites exhibited significant variability in size, ranging from small, localized accumulations to larger, more extensive sites, mostly near the waterbody, previously flooded areas, and erosions. These dumpsites were typically characterized by the absence of containment measures, contributing to uncontrolled waste dispersal and environmental degradation. It was observed that there was an aggregation of what seemed to be “gang members” nearby these dumpsites, sometimes requiring money before collecting information such as pictures and sizes of the dumpsite. Understanding the characteristics of these dumpsites was fundamental to the subsequent analysis.

**Figure 4:**
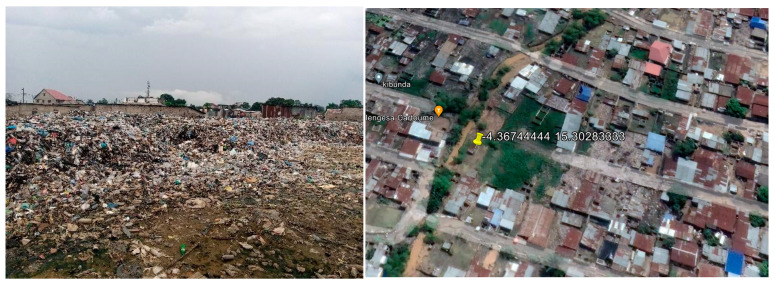
Onsite photo (**left**) and Google earth image (**right**) of dumpsite number 7 in Bumbu.

**Figure 5:**
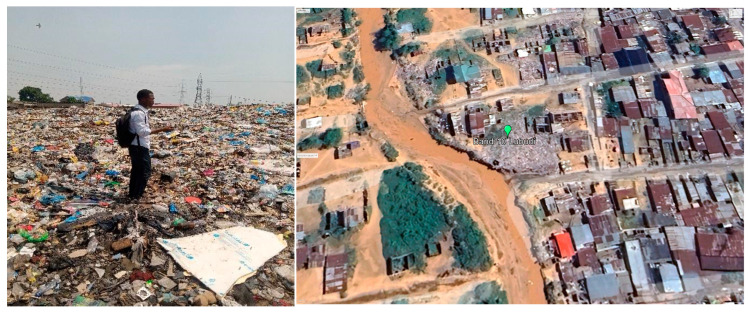
Onsite photo (**left**) and Google earth image (**right**) of dumpsite number 10 in Bandal.

Following Google earth sizing, the largest dumpsites were determined to be number 7 (that revealed to include number 8) for Bumbu, and number 10 for Bandal [Figure 4], [Figure 5]. 

### 3.2. Buffers and Sampling 

**Figure 6:**
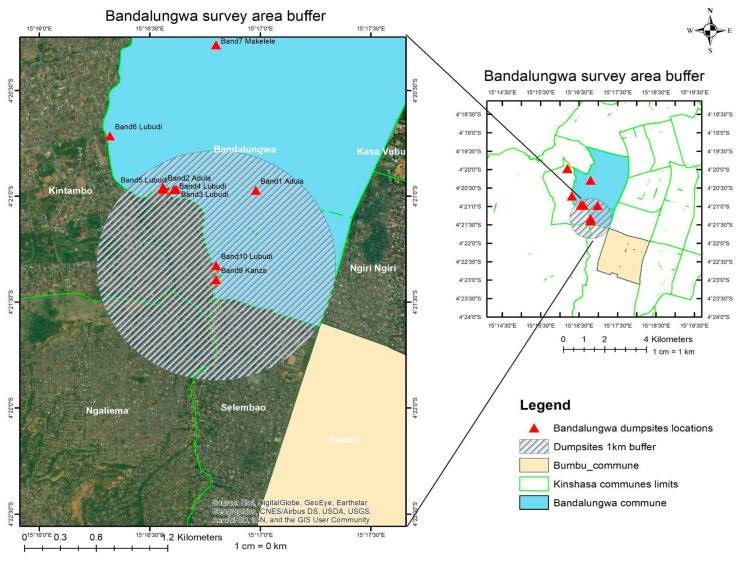
Survey buffer zone in Bandal.

**Figure 7:**
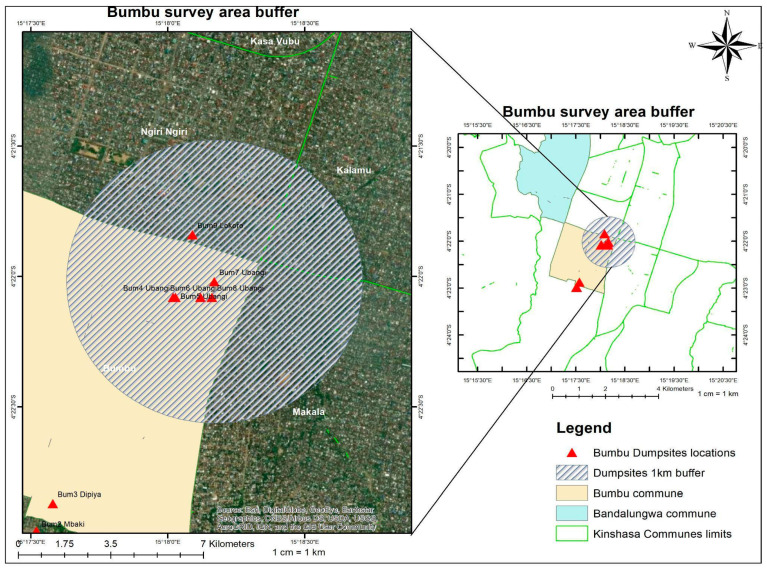
Survey buffer zone in Bumbu.

Following the identification of the largest dumpsite (number 7 for Bumbu, number 10 for Bandal) based on estimated sizes via google Earth pro, a 1 km buffer zone was defined around their periphery to allow sampling of the most exposed households [Figure 6 and Figure 7]. The minimum required sample size for Bandal was calculated to be 379 households, and a total of 380 households were included in the survey. In the case of Bumbu, the minimum sample size was estimated to be 381 households, and the survey covered 391 households.

### 3.3. Survey Results

(a) Socio-demographic data

The socio-demographic data of this survey comprise factors such as Gender, Age Group, Education, Occupation, Work Location, Residency Duration, Smoker Status, and Living with a smoker status. The survey results reflect distinct demographic and lifestyle traits within the surveyed samples of Bandal and Bumbu. In Bumbu, 61% of the surveyed sample comprises females, which is slightly higher than the 58% in Bandal. Moreover, Bumbu displays an overrepresentation of individuals aged 31–40 years, making up 48% of their surveyed sample, while Bandal demonstrates a more evenly distributed age range. Additionally, Bumbu showcases a higher percentage of individuals with a high school education, with 58%, whereas Bandal exhibits more educational diversity. The dominant occupation in both communes’ surveyed samples is entrepreneurship, although Bumbu has a larger proportion of unemployed residents.

Regarding lifestyle and health-related factors, Bumbu has a slightly higher percentage of smokers in its surveyed sample at 16%, compared to Bandal’s 14%. In both communes, most of the surveyed residents do not live with a smoker, although this percentage is slightly higher in Bumbu. The survey results also highlight that the majority of the surveyed residents in both communes have lived there for over 5 years, suggesting a longer exposure time to the potential effects of the dumpsites [Table 4].

(b) Water and food sources

The survey results present a snapshot of the preferences and practices regarding various essential resources within the surveyed samples of Bandal and Bumbu, and the distinct patterns observed are as follows: 

Firstly, when it comes to the main source of drinking water, tap water is the primary choice for residents in both Bandal and Bumbu. However, there is a considerable discrepancy in the reliance on bottled water, with 15% of Bandal’s surveyed sample preferring it compared to only 1% in Bumbu. Additionally, in terms of knowledge about the existence of groundwater sources used in the commune, there is a higher level of uncertainty among Bandal’s surveyed residents (46%) than in Bumbu (7%).

Regarding food sources, both communes predominantly rely on a combination of markets and supermarkets for vegetables. Private gardens also contribute to the vegetable supply, albeit to a lesser extent. Similarly, markets and supermarkets are the primary sources of meat in both communes, with an overwhelming preference for these commercial sources. These insights were measured with the specific intent of assessing whether the population in Bandal and Bumbu might be exposed to sources of malaria and typhoid beyond their proximity to dumpsites. Dumpsites can be potential breeding grounds for disease vectors, and the results from the survey allow for a more comprehensive understanding of the broader environmental and lifestyle factors that could contribute to disease transmission. By examining the choice of drinking water sources and food supply chains, these findings help evaluate the potential risks of malaria and typhoid transmission through avenues other than dumpsites, offering a holistic view of the health landscape in these communes. For instance, reliance on untreated water sources or consumption of locally sourced and unprocessed food may increase the risk of disease transmission, irrespective of the proximity to dumpsites [Table 5].

(c) Reoccurring symptoms

The survey results presented in Table 6 indicate differences in the prevalence of various health symptoms and conditions within the surveyed samples of Bandal and Bumbu. In Bandal, 36% of the surveyed population reported experiencing eye irritation, while in Bumbu, this percentage is higher at 50%. Similarly, other symptoms like nasal irritation, throat pain, and stomach pain are more prevalent in Bumbu compared to Bandal. In contrast, health symptoms such as skin rashes, fatigue, and diarrhea are slightly more common in Bandal. These differences suggest potential variations in the health profiles of the two communes.

The observed health symptoms in the surveyed samples may indeed be linked to exposure to waste open dump sites. For instance, eye and nasal irritation could result from airborne pollutants and irritants often associated with open dumpsites. Similarly, skin rashes and gastrointestinal issues, like stomach pain and diarrhea, can be associated with exposure to contaminated air, water, or soil in the vicinity of dumpsites. However, while these observations are suggestive, further statistical analyses are needed to establish a direct correlation between waste open dumpsite exposure and the prevalence of these health symptoms. Such analyses may involve regression models to determine the strength of the relationship and control for confounding factors, as well as spatial analyses to explore whether proximity to dumpsites significantly affects the likelihood of experiencing these symptoms. Additionally, detailed epidemiological studies, including health records and environmental assessments, would provide a more comprehensive understanding of the potential health risks associated with waste open dumpsite exposure.

(d) Reoccurring Diseases

The survey results, as presented in Table 7, reveal disparities in disease prevalence between the surveyed populations of Bandal and Bumbu. Bumbu exhibits a higher occurrence of certain diseases, particularly typhoid fever and malaria, in comparison to Bandal. Conversely, conditions like asthma, pneumonia, cholera, and food poisoning appear to be less widespread in both communes. 

It is important to note that the higher prevalence of typhoid fever and malaria in Bumbu might be attributed to the fact that Bumbu has larger open dumpsites and lacks formal waste collection services. These open dumpsites can serve as breeding grounds for disease vectors such as mosquitoes and flies, increasing the risk of diseases like malaria and typhoid. Furthermore, inadequate waste management practices in the absence of formal collection services can lead to contamination of water sources, further elevating the likelihood of waterborne diseases. However, to establish a clear and quantifiable link between waste open dumpsite exposure and disease prevalence, in-depth statistical analyses and comprehensive epidemiological studies would be imperative.

(e) Waste management practices

The data from the survey, as shown in Table 8, offers insights into waste management practices and their potential implications for disease prevalence, particularly concerning malaria and typhoid, in both Bandal and Bumbu. Notably, the results indicate that Bumbu exhibits a significantly higher prevalence of relatively younger dumpsites, with a substantial proportion falling into the 1–2 years old and 2–5 years old categories. In contrast, Bandal appears to have a lower prevalence of such younger dumpsites. Additionally, a considerable portion of the surveyed population in Bumbu reported that waste disposal primarily involves burning and dumping, which are methods frequently associated with open dumpsites.

The pronounced contrast in waste disposal practices between Bandal and Bumbu implies a potentially higher risk of disease exposure, specifically for malaria and typhoid, in Bumbu. These waste disposal methods, such as burning, dumping and open dumping, can create environments conducive to disease vectors and environmental contamination, heightening the likelihood of disease transmission. 

A noteworthy aspect of this analysis is that the ages of the dumpsites, as reported by respondents, were cross verified using historical satellite imagery retrieved from Google Earth, which allowed for a retrospective assessment of the existence of these dumpsites five years prior to the survey. In Bandal, the majority of respondents (46%) reported that the dumpsites they were aware of were over 5 years old. And in Bumbu, the majority of respondents (32%) stated that the dumpsites were 2–5 years old. 

**Figure 8:**
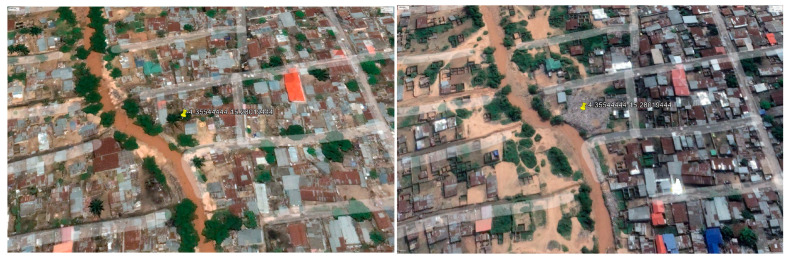
The yellow pinpoint shows dumpsite 10 in Bandal in 2018 (on the **left**) and in 2021 (on the **right**).

**Figure 9:**
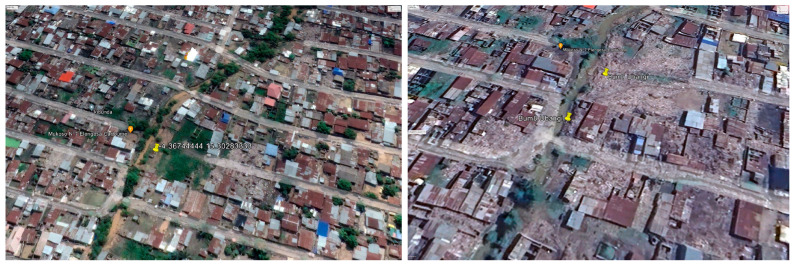
The yellow pinpoint shows dumpsite 7 in Bumbu in 2018 (on the **left**) and in 2021 (on the **right**).

However, the imagery revealed that certain dumpsites, notably dumpsite number 10 in Bandal and dumpsite number 7 in Bumbu (the focus of this buffer study area), did not register on satellite maps before April 2018. However, they began to materialize and become identifiable around April 2021. This suggests that the dumpsites are about 2 years old [Figure 8 and Figure 9]. 

It is important to note that an important percentage of respondents were uncertain of the dumpsite age. In Bandal for instance, 36% of respondents were uncertain about the age of the dumpsites. Additionally, in Bumbu, 25% of respondents in Bumbu were uncertain about the age of the dumpsites.

This finding hints at substantial socio-economic and political transformations in Kinshasa during this period, possibly explaining the sudden appearance and growth of these open dumpsites. Factors such as increased urbanization, population growth, shifts in waste management policies, and economic dynamics may have collectively contributed to the expansion of these informal dumping sites within this time frame.

(f) Healthcare cost

Table 9 presents data on monthly income and monthly medical expenses in Bandal and Bumbu, shedding light on the financial aspects of healthcare within these communities. Notably, in Bumbu, a higher percentage of respondents reported monthly incomes falling within the USD 50–USD 100 and USD 100–USD 500 income brackets compared to Bandal. However, Bandal had a marginally higher proportion of respondents with monthly incomes of more than USD 500. When examining monthly medical expenses, Bumbu residents showed a higher prevalence of spending in the USD 10–USD 50 and USD 50–USD 100 ranges, while Bandal had more respondents in the USD 0–USD 10 range.

The higher prevalence of respondents in Bumbu with higher monthly incomes and medical expenses suggests that Bumbu may carry a higher healthcare burden compared to Bandal. This higher financial healthcare burden in Bumbu could be linked to the fact that this commune is more exposed to open dumpsites and no waste collection services formally implemented by the government, potentially increasing the likelihood of residents contracting diseases like malaria and typhoid, which necessitate medical care. 

Moreover, the large percentage of respondents unwilling to disclose their income may be influenced by socio-cultural factors, as financial discussions can be considered sensitive or private in some cultures. Additionally, it is important to note that both communes may have relatively low-income levels, as indicated by the dominant percentage of respondents with monthly incomes below USD 100. These low-income levels are reflective of the broader economic conditions in the Democratic Republic of Congo, where a significant portion of the population lives on very limited means. The average daily income in the country, often much lower than USD 1 [58], underscores the financial challenges faced by many individuals in Bandal and Bumbu, which can significantly impact their ability to afford healthcare.

(g) Public awareness and opinions

Table 10 provides insights into public awareness and opinions related to waste management in Bandal and Bumbu. Notably, in both communes, the majority of respondents recognized a linkage between symptoms and diseases with open dumpsites, with Bumbu showing a slightly higher level of awareness. A significant proportion of respondents in both communes found open dumpsites to be a nuisance, with the majority acknowledging this issue.

Regarding information sources on waste management, television emerged as a prominent source of information for residents in both communes, with radio and the internet also playing a role, albeit to a lesser extent. Additionally, a substantial number of respondents in Bumbu relied on television and the internet as their main sources of information.

The portion of respondents who indicated that they were not informed about waste management practices is an important aspect to consider in the context of public awareness and attitudes toward waste sustainability. In both Bandal and Bumbu, a notable percentage of respondents fell into this category, with a significantly higher proportion in Bumbu (56%).

This lack of information suggests that there is a segment of the population in these communes that may not have access to, or engagement with, mainstream information sources. It could be due to various reasons, such as limited access to media or lower levels of education. These individuals may not be fully aware of the potential health and environmental impacts of poor waste management or the importance of sustainable waste practices.

Addressing this information gap is critical for achieving a more comprehensive and inclusive approach to waste management education and awareness. 

The awareness and information sources regarding waste management are crucial elements in fostering a more responsible and sustainable attitude toward waste. A well-informed public is more likely to understand the environmental and health impacts of waste mismanagement, which can lead to increased support for sustainable waste practices. Additionally, public awareness can put pressure on central governments and municipalities to implement better waste management policies and practices. The information flow provided by various sources can help bridge the knowledge gap and educate the public about the importance of proper waste disposal, recycling, and the reduction of waste-related health risks.

The survey incorporated an open-ended question that encouraged respondents to provide their public opinions regarding waste management in their communes. This open question serves a crucial purpose in the survey, as it allows respondents to express their views and insights in their own words. Open questions are valuable in research because they enable participants to share unfiltered, authentic perspectives, raising issues or providing feedback that may not have been anticipated in closed-ended questions. 

In Bandal, the opinions were stated as follows:

- Frustration with current waste management;
- Call for public discussions and awareness campaigns;
- Demands for government responsibility;
- Pleas for authorities to listen to the people;
- Desire for community involvement in finding solutions;
- Emphasis on sanitation and proper waste disposal;
- Calls for public trash bins and maintenance;
- Need for drainage system cleaning;
- Acknowledgment of the community’s role in addressing waste;
- Requests to reinstate hygiene services;
- Suggestions for public trash bin creation;
- Calls for community-oriented solutions from authorities;
- Recognition of the community’s role in taking responsibility;
- Importance of organized sanitation services;
- Calls to revamp the hygiene service;
- Demand for municipal authorities to combat waste pollution;
- Desire for improved city waste management;
- Importance of government support and resources;
- Pleas for public trash bins placement and government action;
- Concerns about a lack of government projects;
- Belief in government involvement for lasting solutions;
- Need for public awareness of cleanliness dangers;
- Calls for suitable waste disposal locations;
- Requests for relocation until waste issues are resolved;
- Focus on resolving flooding issues as a root cause of waste pollution;
- Suggestions for river-side facilities;
- Increase in waste collection frequency;
- Requests for dumpsite relocation to reduce health risks;
- Plea to remove garbage piles due to mosquito infestations;
- Proposals for creating waste management sites;
- Need for more sanitation workers;
- Pleas for government assistance in sustainable waste management;
- Acknowledgment of local NGOs and environmental organizations;
- Suggestions for recycling and reusing waste;
- Concerns about mosquito infestations.

The community’s public opinion on waste management in Bandal emphasizes frustration with the current situation, demands government responsibility, and calls for community involvement, sanitation improvement, and public trash bins, while acknowledging the importance of public awareness, sanitation workers, and support from authorities and organizations.

In Bumbu, opinions were stated as follows:

- Dredging the river to prevent flooding;
- Criticism of poor waste management in the community and a call for hygiene service renewal;
- Implementation of waste collection vehicles for daily pickups;
- Suggestions for recycling or transforming waste, possibly by establishing a recycling facilities;
- A loss of trust in the government due to unfulfilled promises, with a demand for the reinstatement of hygiene services;
- A lack of information on waste management;
- The need for flood control measures to address waste issues;
- Building along the banks of the Kalamu River and cleaning the river, as well as prohibiting waste dumping;
- Initiating effective awareness campaigns and providing necessary resources for proper waste management;
- Stop using waste as a means to combat flooding in the neighborhood and requesting government support for alternative flood control methods;
- Relocating the landfill and cleaning the Kalamu River;
- Frustration with unfulfilled government promises and a call for unity;
- The importance of each community developing a clean waste management plan;
- A call for authorities to take responsibility;
- A reference to Article 53 of the constitution and its lack of implementation;
- Emphasis on the need for public awareness and education;
- Proposing a hierarchy for waste management across the city and community awareness;
- Suggesting organizing regular clean-up events throughout the city and providing waste treatment facilities for sustainable management;
- Placement of public trash bins on every street corner and a large communal bin in Bumbu Commune;
- Identifying the root cause of illegal dumping in the neighborhood and advocating for riverbank construction;
- Acknowledging that responsibility for waste management is a shared endeavor.

The public opinion on waste management in Bumbu emphasizes the need to address flooding issues caused by waste accumulation. Residents call for the relocation of trash bins and express dissatisfaction with the current waste management system, urging the renewal of hygiene services. Some suggest the use of waste collection vehicles and encourage recycling efforts, either by establishing recycling facilities or returning to “traditional methods” of recycling. There is a prevailing sense of frustration with unfulfilled government promises and a call for trust restoration through the reinstatement of hygiene services. Residents stress the importance of public awareness, proposing organized clean-up events and the establishment of waste treatment facilities for sustainable management. The community highlights the shared responsibility for waste management and emphasizes the need for flood control measures, such as riverbank construction and prohibition of waste dumping.

(h) Survey results analysis

The statistical analysis performed in R Studio aimed to test the study’s hypotheses regarding the correlation between waste management practices in Bandal and Bumbu and the prevalence of malaria and typhoid. Additionally, the study aimed to investigate if the prevalence of these illnesses differed between the two communes.

The analysis began with the calculation of the statistical prevalence of malaria and typhoid, comparing the two communes. The results indicated that both malaria and typhoid were significantly prevalent in both communes, with Bumbu showing a higher prevalence of these illnesses compared to Bandal. The prevalence of malaria was lower in Bandal (92.6%) compared to Bumbu (97.2%), as indicated by a chi-squared test (X^2^) with a *p*-value of less than 2.2 × 10^−16^. Similarly, the prevalence of typhoid was notably elevated in Bumbu (91.3%) compared to Bandal (77.4%), with a highly significant X^2^ result (*p* < 2.2 × 10^−16^).

These disparities in disease prevalence could be attributed to several factors. One key factor may be the difference in waste management practices and the presence of open dumpsites in each commune. Bumbu’s higher prevalence of these diseases might be associated with the presence of larger open dumpsites, which can serve as breeding grounds for disease vectors and environmental contamination. Additionally, the absence of proper waste collection services in Bumbu could contribute to the increased prevalence of diseases as it may lead to the improper disposal of waste, further exposing the population to health risks.

The chi-squared test (X^2^) was an essential statistical analysis in this study as it helped determine whether the observed differences in disease prevalence between Bandal and Bumbu were statistically significant. The *p*-value associated with the X^2^ test indicates the probability of obtaining such results if there was no actual association between the variables. In this context, the *p*-value being less than 2.2 × 10^−16^ suggests a highly significant association between the communes and disease prevalence, which supports the notion that these differences are not due to chance [Table 11].

Subsequent logistic regression analyses were conducted to explore further correlations in this study. Notably, the test was run to analyze the correlation between the prevalence of typhoid and malaria with self-collection practices of waste in Bandal and Bumbu, and with the presence/absence of formal waste collection implementation.

In Bandal, it was found that individuals who practiced self-collection of waste had a notably higher likelihood of contracting typhoid, with an odds ratio (OR) of 4.834 and an extremely low *p*-value of 0.00001. Conversely, in Bumbu, the practice of self-collection did not appear to have a statistically significant association with typhoid, as reflected by a lower OR of 0.441 and a *p*-value of 0.08. Even though Bumbu has a higher prevalence of self-collection, other factors might mitigate the risk of typhoid. The absence of formal waste collection services in Bumbu could lead to different waste management behaviors, which may not be directly linked to an increased risk of typhoid. It is important to note that the *p*-value of 0.08, while higher than in Bandal, still suggests that there may be a potential link that did not reach statistical significance in this case.

Furthermore, the presence of formal waste collection services demonstrated that in Bandal, the presence of these services was linked to a reduced likelihood of typhoid, with an OR of 0.206 and an extremely low *p*-value of 0.00001. However, in Bumbu, the absence of waste collection services was associated with a notably higher likelihood of typhoid, with an OR of 2.268 and a *p*-value of 0.08.

These findings suggest that waste services may play a protective role against typhoid in Bandal. Conversely, in Bumbu, the limited access to waste services may expose the population to a higher risk of contracting typhoid.

For malaria, the results suggest that, in Bandal, individuals who practice self-collection of waste have a significantly higher risk of contracting malaria, as evidenced by a substantial odds ratio (OR = 6.813) and a low *p*-value (0.009). This means that the presence of self-collection is strongly associated with an increased likelihood of malaria in this commune. The low *p*-value indicates strong statistical evidence supporting this link. On the other hand, the presence of formal waste collection services in Bandal is found to be protective against malaria, indicated by a low OR (0.147) and a significant *p*-value (0.009). These results imply that individuals in Bandal who rely on formal waste services might have a lower risk of contracting malaria because these services likely promote more controlled and hygienic waste disposal practices.

In contrast, in Bumbu, the practice of self-collection does not demonstrate a statistically significant link to malaria, as reflected by a lower OR (0.443) and a non-significant *p*-value (0.3). This suggests that self-collection of waste is not significantly associated with increased malaria prevalence in Bumbu. Moreover, the presence of formal waste collection services also does not exhibit a significant connection with malaria prevalence in Bumbu, as indicated by an OR of 2.259 and a non-significant *p*-value of 0.3. One crucial aspect to consider is that there are no formal waste collection services in Bumbu in the first place. Therefore, the non-significant correlation between malaria prevalence and waste collection services can be attributed to the absence of formal services.

In summary, these findings overall suggest that waste services may play a protective role against diseases in Bandal. Conversely, in Bumbu, the limited access to waste services may expose the population to a higher risk of contracting typhoid and malaria. The differences in results between the two communes could be attributed to variations in waste management behaviors and infrastructure [Table 12].

Furthermore, correlation analysis between the prevalence of malaria and typhoid and specific waste management practices such as burning, burying and open dumping were run separately for Bumbu and Bandal. 

The results showed that in Bumbu, the OR for typhoid associated with burning was 0.934, and the *p*-value was 0.866, implying that burning waste does not significantly correlate with typhoid in this area. Similarly, in Bandal, the OR for burning was 0.344, with a *p*-value of 0.089, suggesting a non-significant trend regarding this practice. The results for burying in both communes demonstrated ORs of 1.45 (Bumbu) and 1.507 (Bandal), with *p*-values of 0.623 and 0.6, respectively. These findings indicate that burying waste is not significantly linked to typhoid in either commune. Finally, open dumping in both communes showed ORs of 0.952 (Bumbu) and 1.543 (Bandal), with *p*-values of 0.898 and 0.426, respectively, further confirming that open dumping is not significantly associated with typhoid prevalence in these areas. The results suggest that the waste management practices assessed (burning, burying, and open dumping) do not exhibit statistically significant connections with typhoid prevalence in Bandal and Bumbu. These practices may not be the primary factors contributing to the prevalence of typhoid in these communes, highlighting the complexity of disease dynamics and other potential influential factors that need to be considered.

For malaria, results showed that burning waste in Bandal exhibited an exceptionally high OR of 5681313 associated with burning waste. Despite this striking OR, the remarkably high *p*-value of 0.995 implies that this association lacks statistical significance. In Bumbu, the OR is considerably lower at 3.662, with a *p*-value of 0.224, indicating a non-significant connection. These findings collectively suggest that burning waste does not have a statistically significant impact on the prevalence of malaria in either commune.

In the case of burying waste, both Bandal and Bumbu share similar patterns of extremely high ORs with *p*-values of 0.996 and 0.993, respectively, emphasizing non-significant associations. These results indicate that burying waste does not exhibit significant links with the prevalence of malaria in either commune, despite the remarkably high ORs. It is important to note that the non-significant *p*-values reinforce the absence of a statistical connection between burying waste and malaria prevalence, highlighting that other factors are likely more influential in determining the disease’s spread in these areas. The ORs for open dumping paint a clear picture of non-significant associations with malaria in both communes. In Bandal, the extremely low OR (0.00000005) and a non-significant *p*-value of 0.994 indicate no substantial link between open dumping and malaria. In Bumbu, the low OR of 0.18 and a non-significant *p*-value of 0.1 further underline that open dumping of waste does not significantly contribute to malaria prevalence. The results suggest that the waste management practice of open dumping is unlikely to be a major factor in the spread of malaria in these areas. In summary, the analysis demonstrates that the chosen waste management practices, including burning, burying, and open dumping, do not exhibit statistically significant associations with the prevalence of malaria in Bandal and Bumbu. 

The anticipated positive correlation between the prevalence of malaria and open dumping of waste may not have materialized due to an indirect relationship. In this scenario, the primary link is likely to be through the breeding grounds created for the Anopheles mosquito, a known malaria vector. Stagnant water, a common consequence of waste deposition, serves as an ideal breeding site for these mosquitoes. Specifically, certain waste items like PET bottles can inadvertently become breeding nests when they collect rainwater, providing an environment conducive to mosquito reproduction.

In essence, while open dumping of waste may not directly lead to malaria transmission, it indirectly contributes by providing breeding grounds for malaria-carrying mosquitoes. Therefore, the presence of waste, especially in the form of discarded items that collect rainwater, indirectly fosters the propagation of malaria vectors, ultimately contributing to the higher prevalence of the disease [Table 13].

Lastly, the analysis explored the potential correlation between waste management practices such as waste burning, and symptoms such as eyes irritation and nasal irritation reported by respondents. 

Results showed that for nasal irritation in Bumbu, the estimate is −0.494, with a significant *p*-value of 0.041. The odds ratio (OR) is 0.610, indicating that individuals exposed to waste burning practices in Bumbu have a 0.610 times lower likelihood of experiencing nasal irritation. For eyes irritation, the estimate is 0.387, with a *p*-value of 0.094. The OR is 1.473, suggesting that individuals exposed to waste burning practices in Bumbu have a 1.473 times higher likelihood of experiencing eye irritation. The presence of a smoker in the household or their working environment is estimated to have a significant positive effect with an OR of 1.722, indicating that living with a smoker is associated with a 1.722 times higher likelihood of experiencing irritation symptoms. 

For nasal irritation in Bandal, the estimate is −0.639, but the *p*-value is not significant (*p* = 0.194), suggesting that waste burning practices in Bandal are not significantly associated with nasal irritation. The OR is 0.528, implying a 0.528 times lower likelihood of nasal irritation among those exposed to waste burning practices. For eyes irritation, the estimate is 0.266, with a non-significant *p*-value of 0.569. The OR is 1.304, indicating that individuals exposed to waste burning practices in Bandal have a 1.304 times higher likelihood of experiencing eye irritation compared to those not exposed. The presence of a smoker in the household or the work environment is estimated to have a non-significant effect (*p* = 0.239) with an OR of 0.563, implying a 0.563 times lower likelihood of experiencing irritation symptoms when living with a smoker.

The analysis suggests that waste burning practices in Bumbu are associated with a lower likelihood of nasal irritation but a higher likelihood of eye irritation. Living with a smoker is associated with an increased likelihood of irritation symptoms in Bumbu. However, in Bandal, waste burning practices are not significantly associated with nasal irritation, and the relationship with eye irritation is not conclusive. Living with a smoker does not show a significant association with irritation symptoms in Bandal. The results indicate the complex and varied nature of these associations, emphasizing the need to consider multiple factors when examining the impact of waste management practices on reported symptoms [Table 14].

## 4. Conclusions

This study explored the impact of waste management practices on health outcomes, specifically focusing on the prevalence of malaria and typhoid in Kinshasa’s Bandalungwa (Bandal) and Bumbu communes. The research revealed significant disparities in waste management infrastructure, the spatial distribution of dumpsites, and the prevalence of diseases. The correlation between waste management practices and health outcomes is both direct and indirect, highlighting the critical need for improved waste management services, particularly in underserved areas like Bumbu. 

The analysis demonstrated that both malaria and typhoid are significantly more prevalent in Bumbu compared to Bandal. For instance, malaria prevalence was higher in Bumbu (97.2%) than in Bandal (92.6%), and similarly, typhoid prevalence was also more pronounced in Bumbu (91.3%) compared to Bandal (77.4%). These disparities can be attributed to the absence of formal waste collection services and the presence of larger open dumpsites in Bumbu, which serve as breeding grounds for disease vectors like mosquitoes. The statistical significance of these differences (*p* < 2.2 × 10^−16^) underscores the strong association between poor waste management and the higher prevalence of these diseases. The results also showed that formal waste collection services in Bandal are associated with a reduced likelihood of contracting malaria and typhoid. Logistic regression analysis revealed that self-collection of waste significantly increases the risk of these diseases in Bandal, with typhoid (OR = 4.834, *p* = 0.00001) and malaria (OR = 6.813, *p* = 0.009) both showing strong positive correlations. In contrast, Bumbu, where formal waste services are absent, did not exhibit statistically significant correlations between self-collection and disease prevalence, likely due to the already high exposure to open dumpsites. These findings align with other studies highlighting the public health impacts of inadequate waste management. For example, previous works have shown a clear connection between waste exposure and increased disease transmission, respiratory problems, and other health risks [17,30,32,33,34,49,50,51]. Studies across different regions emphasize the negative outcomes from proximity to waste dumpsites, mirroring the situation observed in Bumbu. For instance, Vinti et al. (2021) documented a higher prevalence of neonatal complications and respiratory diseases among residents near municipal solid waste (MSW) sites. Similar findings were observed by Giovanni et al. (2023), who emphasized the elevated risks of vector-borne diseases and direct exposure to hazardous contaminants. These studies provide a global context to the Bumbu case, where inadequate waste management is linked to health risks like respiratory and vector-borne diseases.

However, as seen in Bandal, areas with better waste management infrastructure tend to have lower disease prevalence. This trend supports the argument that waste management acts as a protective factor against environmental health risks. The correlation between open dumping and malaria observed in Bumbu is consistent with Afra Tanjim et al. (2023), who highlighted the role of stagnant water in dumped waste as breeding grounds for mosquitoes, reinforcing the importance of waste management in controlling vector-borne diseases. In contrast to the findings from Nairobi and Accra mentioned earlier, where proximity to dumpsites was strongly associated with vector-borne diseases like malaria, the current study did not find a statistically significant correlation. However, stagnant water in improperly discarded waste still presents a potential risk. This echoes the work of Singh et al. (2020), who found that health outcomes such as respiratory illnesses and stomach problems were significantly higher among those exposed to dumpsites.

The study also uncovered significant economic consequences associated with poor waste management. In Bumbu, residents face higher healthcare costs, potentially due to the higher disease burden. This economic strain further exacerbates the cycle of poverty and poor health, a pattern observed in other urban areas with inadequate waste management infrastructure. The findings suggest that improving waste management services in areas like Bumbu could lead to not only better health outcomes but also economic benefits by reducing healthcare expenses.

Based on the findings, this research suggests that policymakers should prioritize Solid Waste Management services to underserved communities, enforce stricter waste disposal regulations, and invest in modern waste treatment infrastructure. Public health campaigns should raise awareness about the dangers of improper waste disposal, while public health surveillance must be strengthened to monitor disease outbreaks linked to waste exposure. Additionally, integrating waste management into urban planning and targeting disease prevention programs could further reduce health risks and improve the overall well-being of residents. These actions are essential for creating a healthier and more sustainable urban environment.

Future research should address several key gaps. One important direction is the need for longitudinal studies to track changes in health outcomes over time as waste management practices improve. Additionally, more granular data on waste exposure, such as the frequency and duration of contact with waste dumpsites, could enhance understanding of the health impacts. A critical focus for future research should be the standardization of methodologies to clearly define the links between waste exposure and health outcomes. This would ensure that the causal agents, such as specific contaminants or vectors, are more precisely identified and their effects better understood. Lastly, exploring the socioeconomic dynamics of waste management, including the costs and benefits of formal waste services, could provide valuable insights for policymakers to develop sustainable, health-focused waste management solutions.

## 5. Limitations 

It is imperative to address the following limitations of the study:

- Data Collection: The study’s reliance on self-reported survey data opens the possibility of recall bias and the influence of respondents’ subjective perceptions. To enhance data quality, future research could incorporate additional data sources, such as health records and comprehensive environmental assessments.
- Correlation vs. Causation: The study successfully identifies correlations between waste management practices and disease (malaria and typhoid) prevalence. However, it is important to note that it does not establish causation. The absence of a formal methodological framework for examining the direct correlation between exposure to waste dumpsites and health outcomes makes it difficult to definitively attribute illnesses solely to waste exposure. Many other factors, such as individual behaviors and healthcare accessibility, and many more, could also influence the prevalence of these diseases. To enhance the rigor of future research, a more comprehensive methodology might involve tracking the long-term trends in disease prevalence (using data from health centers and more formal sources instead of self-reported diseases) alongside monitoring the changes in open dumpsites, including their size and numbers, in the target areas. This approach would provide a more holistic understanding of the complex factors at play and better inform strategies to mitigate health risks associated with waste management practices.
- Socio-Cultural Factors: The study recognizes that socio-cultural factors can significantly influence public awareness and individuals’ willingness to disclose financial information. Future research should consider delving deeper into economic aspects of these health outcomes to gain a more profound understanding of waste management practices and the subsequent healthcare’s financial burden.
- Policy and Infrastructure: The study does not delve into the broader policy and infrastructure issues that can impact waste management practices. It is crucial to emphasize that understanding the regulatory environment and local infrastructure constraints is essential for formulating well-informed policy recommendations that address waste management at its core.

## Figures and Tables

**Table 1:** Researchers’ key findings on public health implications from exposure to waste dumpsites.

Author(s)	Year of Publication	Key Findings
Vinti G, Bauza V, Clasen T, Medlicott K, Tudor T, Zurbrügg C, Vaccari M.	2021	The findings suggested an increased risk of adverse birth and neonatal outcomes for residents near each type of MSW site. There was evidence of increased risk of mortality, respiratory diseases, and negative mental health effects associated with residing near landfills.
Tomita A, Cuadros DF, Burns JK, Tanser F, Slotow R.	2020	Residing within 5 km of a waste site was found to be significantly associated with several health conditions.
Giovanni Vinti, Valerie Bauza, Thomas Clasen, Terry Tudor, Christian Zurbrügg, Mentore Vaccari	2023	The analysis identifies that dumpsites and uncontrolled burying of solid waste pose the highest health risks, leading to a very high or high risk of infectious and vector-borne diseases. Additionally, there is a significant risk of inhalation, ingestion, or dermal contact with contaminants during waste disposal in dumpsites, open burning, and reuse of waste from dumpsites as compost.
Afra Tanjim Shammi, Nazia Hassan, Md Rony Golder, Hriday Molla, Shikder Saiful Islam	2023	This study in Bangladesh revealed that participants, predominantly with low income and residing over 10 years in the community, identified odor from the dumpsites as their primary concern (74%). Approximately 18% reported the severe impact of smoke from burning waste on their health. Health problems included dysentery, diarrhea, pulmonary diseases, asthma, and allergies. Proximity to dumpsites showed a statistically significant association with health problems, water pollution, and unpleasant odor.
Joseph Omeiza Alao	2023	This study assesses the impacts of dumpsite leachate on soil and groundwater quality through geophysical and physicochemical water analysis methods. Results indicated the presence of heavy metals, soft subsoil, and low resistivity values, aligning with the compromised soil and aquifer systems influenced by leachate plumes.
Singh, S. K., Chokhandre, P., Salve, P. S., & Rajak, R.	2020	The exposed group to dumpsites in this study shows a higher prevalence of selected morbidities, such as respiratory illness, eye irritation, and stomach problems, compared to the non-exposed group. Multivariate analysis confirms that respondents from the exposed group are significantly more likely to suffer from respiratory illness, eye infection, and stomach problems.
Ogunrinola, I. O., & Adepegba, E. O.	2012	The results indicate that pollution variables significantly impact the health status and labor supply of the sampled respondents.
Tukura, E. D., Ojeh, V. N., Philip, A. H., & Ayuba, A.	2018	The findings indicate that residences within certain distances of dumpsites are at risk of dumpsite-related diseases.

**Table 3:** Surveyed dumpsites’ geo data.

Commune	Altitude (m)	Latitude	Longitude	Volume (m^3^) *	Area (km²) **	Area (ha) **
Bumbu	320	4.38294444	15.29194444	1233	0.01	0.8
	320	4.38308333	15.29202778	700		
	317	4.38119444	15.29297222	650	0	0.13
	306	4.36805556	15.30033333	75	-	-
	303	4.36769444	15.30047222	6.4	-	-
	304	4.36761111	15.30158333	21	-	-
	298	4.36744444	15.30283333	18,750	0.03	2.76 ha
	297	4.36766667	15.30269444	20,240		
	301	4.36441667	15.30150000	171.5	-	-
Bandal	296	4.34952778	15.28275000	67.5	-	-
	293	4.34927778	15.27638889	630	-	0.06
	289	4.34944444	15.27658333	211.2		
	287	4.34947222	15.27683333	224	-	-
	285	4.34944444	15.27558333	262.5	0	0.05
	287	4.34525000	15.27188889	49.14	0	0.04
	294	4.33808333	15.27955556	100	0	0.04
	287	4.33277778	15.26980556	10,500	0	1.19
	291	4.35655556	15.28047222	374	0	0.03
	293	4.35544444	15.28019444	825	0	0.09

* Estimated using measuring tape during field survey. ** Estimated using Google Earth pro satellite imagery.

**Table 4:** Demographic and lifestyle characteristics in Bandal and Bumbu.

Independent Variables	Dependent Variables	Bandal	Bumbu
Gender	Female	58%	61%
	Male	42%	39%
Age group	18–30 years old	34%	27%
	31–40 years old	41%	48%
	More than 40 years old	25%	25%
Education	Primary school	1%	4%
	Middle school	6%	12%
	High School	37%	58%
	Bachelor	21%	17%
	Master’s	27%	4%
	Post master’s	8%	4%
Occupation	Employee	18%	18%
	entrepreneur	52%	49%
	Retired	2%	3%
	Student	14%	6%
	Unemployed	15%	24%
Work location	Within commune	44%	37%
	Out of the commune	32%	34%
	Non-Applicable	24%	29%
Residency duration in the commune	0–1 year	8%	9%
	1–2 years	11%	15%
	2–5 years	19%	17%
	Over 5 years	62%	60%
Smoker	Yes	14%	16%
	No	86%	84%
Living with smoker	Yes	41%	43%
	No	59%	57%

**Table 5:** Water and food sources in Bandal and Bumbu.

Independent Variables	Dependent Variables	Bandal	Bumbu
Main source of drinking water	Bottled water	15%	1%
	Groundwater	1%	1%
	Tap water	82%	98%
	River	0%	0%
Knowledge of an existing groundwater source, frequently used in the commune	Uncertain	46%	7%
	No	42%	54%
	Yes	13%	39%
Main source of vegetables	Markets	1%	0%
	Supermarkets	0%	0%
	Markets and supermarkets	97%	98%
	Private garden	2%	2%
Main source of meat	Markets and supermarkets	99%	100%
	Farm	1%	0%

**Table 6:** Health symptoms and conditions in Bandal and Bumbu.

Independent Variables	Dependent Variables	Bandal	Bumbu
Eyes irritation	Yes	36%	50%
	No	64%	50%
Skin rashes	Yes	29%	32%
	No	71%	68%
Nasal irritation	Yes	52%	69%
	No	48%	31%
Headaches	Yes	84%	88%
	No	16%	12%
Fatigue	Yes	19%	26%
	No	81%	74%
Throat pain	Yes	31%	36%
	No	69%	64%
Stomach pain	Yes	42%	51%
	No	58%	49%
Diarrhea	Yes	30%	49%
	No	70%	51%

**Table 7:** Disease prevalence in Bandal and Bumbu.

Independent Variables	Dependent Variables	Bandal	Bumbu
Asthma	Yes	10%	18%
	No	90%	82%
Pneumonia	Yes	7%	4%
	No	93%	96%
Typhoid fever	Yes	77%	91%
	No	23%	9%
Cholera	Yes	3%	1%
	No	97%	99%
Food poisoning	Yes	10%	2%
	No	90%	98%
Malaria	Yes	93%	97%
	No	7%	3%

**Table 8:** Waste management practices in Bandal and Bumbu.

Independent Variables	Dependent Variables	Bandal	Bumbu
Dumpsite age	0–1-year-old	2%	4%
	1–2 years old	6%	15%
	2–5 years old	11%	32%
	Over 5 years old	46%	24%
	Uncertain	36%	25%
Waste collection	Private services	63%	30%
	Public services	2%	0%
	The household	32%	61%
	Uncertain	3%	8%
Waste disposal	landfilled	0%	0%
	Buried	9%	8%
	Burned	6%	29%
	Dumped	36%	57%
	Uncertain	50%	5%

**Table 9:** Financial aspects of healthcare in Bandal and Bumbu.

Independent Variables	Dependent Variables ($ = USD)	Bandal	Bumbu
Monthly income ($ = USD)	$0–$10	0%	0%
	$10–$50	1%	1%
	$50–$100	8%	17%
	$100–$500	7%	9%
	More than $500	2%	0%
	Unwilling to disclose	57%	65%
	Uncertain	25%	7%
Monthly medical expenses ($ = USD)	$0–$10	4%	3%
	$10–$50	16%	54%
	$50–$100	7%	12%
	$100–$500	1%	1%
	More than $500	0%	0%
	Unwilling to disclose	22%	4%
	Uncertain	49%	26%

**Table 10:** Public awareness on waste management in Bandal and Bumbu.

Independent Variables	Dependent Variables	Bandal	Bumbu
Symptom linkages with dumpsites	No	1%	1%
	Yes	80%	93%
	Uncertain	19%	6%
Disease linkages with dumpsites	No	1%	0%
	Yes	95%	99%
	Uncertain	4%	1%
Dumpsites constituting a nuisance	No	6%	3%
	Yes	91%	96%
	Uncertain	3%	0%
Waste management’s main information sources	General public	1%	2%
	Television	31%	29%
	Radio	0%	1%
	Internet	1%	1%
	University	0%	0%
	Waste pickers	0%	0%
	Television, radio	0%	1%
	Television, internet	29%	4%
	Television, NGOs	1%	0%
	Newspapers and posters	2%	1%
	Internet, newspapers and posters	0%	0%
	Television, internet, newspapers and posters	19%	5%
	Uninformed	16%	56%

**Table 11:** Statistical prevalence of malaria and typhoid in Bandal and Bumbu.

	Malaria Prevalence	Typhoid Prevalence	*p*-Value
Bandal	92.6%	77.4%	<2.2 × 10^−16^
Bumbu	97.2%	91.3%	<2.2 × 10^−16^

**Table 12:** Typhoid and malaria correlation with waste services and self-collection in Bandal and Bumbu.

	Typhoid (Self-Collection)	Typhoid (Waste Services)	Malaria (Self-Collection)	Malaria (Waste Services)
Bandal	OR = 4.834, *p* = 0.00001	OR = 0.206, *p* = 0.00001	OR = 6.813, *p* = 0.009	OR = 0.147, *p* = 0.009
Bumbu	OR = 0.441, *p* = 0.08	OR = 2.268, *p* = 0.08	OR = 0.443, *p* = 0.3	OR = 2.259, *p* = 0.3

**Table 13:** Typhoid and malaria correlation with waste management practices in Bandal and Bumbu.

Waste Management Practice	Commune	Typhoid OR	Typhoid *p*-Value	Malaria OR	Malaria *p*-Value
Burning	Bandal	0.344	0.089	5681313	0.995
	Bumbu	0.934	0.866	3.662	0.224
Burying	Bandal	1.507	0.6	1653940	0.996
	Bumbu	1.45	0.623	8573619	0.993
Open Dumping	Bandal	1.543	0.426	0.00000005	0.994
	Bumbu	0.952	0.898	0.18	0.1

**Table 14:** Symptoms correlation with waste management practices in Bandal and Bumbu.

Variable	Pr(z) (Bumbu)	Odds Ratio (Bumbu)	Pr(z) (Bandal)	Odds Ratio (Bandal)
Nasal Irritation	0.0414	0.610	0.194	0.528
Eyes Irritation	0.0944	1.473	0.5699	1.304
Living with a Smoker	0.0175	1.722	0.2394	0.563

## Data Availability

Date will be made available upon request.
